# Reliable information for cancer control in Cali, Colombia

**DOI:** 10.25100/cm.v49i1.3689

**Published:** 2018-03-30

**Authors:** Luis Eduardo Bravo, Luz Stella García, Paola Collazos, Edwin Carrascal, Oscar Ramírez, Tito Collazos, Armando Cortés, Marcela Nuñez, Erquinovaldo Millan

**Affiliations:** 1 Registro Poblacional de Cáncer de Cali. Cali, Colombia; 2 Departamento de Patología, Facultad de Salud, Universidad del Valle, Cali, Colombia.; 3 Fundación Pohema. Cali, Colombia; 4 Sistema de Vigilancia Epidemiologica de Cáncer Pediátrico (VIGICANCER), Cali, Colombia.; 5 Secretaria de Salud Pública Municipal de Cali, Cali, Colombia.

**Keywords:** Cancer registry, incidence, mortality, survival, methods, Cali, Colombia, Registro de cáncer, incidencia, mortalidad, supervivencia, métodos, Cali, Colombia

## Abstract

**Background:**

The Cali Population Cancer Registry (RPCC) has been in continuous operation since 1962 with the objective of producing valid statistics on the incidence of cancer, its patterns, trends and survival rates.

**Methods:**

During the period 2008-2012, 23,046 new cases were registered and during 2011-2015 there were 12,761 cancer deaths. The trend of the rates was described with the APC average annual change rate and with the Joinpoint analysis. We analyzed the individual data of 38,671 adults (15-99 years) diagnosed with cancer between 1995-2009, and we calculated the standardized net survival by age for the 14 most common cancer body sites, using the Pohar-Perme method.

**Results:**

Prostate and breast cancer were the first cause of cancer morbidity. The incidence rates in these were susceptible to early detection, tumors stabilized after decades of growth, while an increase in the incidence of colon cancer and papillary thyroid carcinoma was observed. The incidence rates of cervical and stomach cancer and conditions related to infectious agents decreased, although the number of absolute cases increased, due to the growth and aging of the population. Gastric cancer was responsible for the highest number of cancer related deaths. The types of cancer related to tobacco consumption (lung, oral cavity, esophagus, pancreas, urinary bladder) showed low numbers and a tendency to decrease. During the period 2000-2004, the 5-year net survival improved for cancers of the breast, cervix, prostate, melanoma and thyroid, although in the period 2005-2009 a stagnation was observed. In stomach, liver and lung cancer, the 5-year net survival was less than 15%. The 5-year overall survival in children was 51.0% (95% CI: 47.5, 54.3) and in adolescents 44.6% (95% CI: 36.0, 52.8).

**Comment:**

RPCC has been an advisor to the Colombian government in the evaluation of CPRs in the country and its data has contributed significantly to different aspects of cancer control in Colombia.

## Resumen

**Antecedentes:**

El Registro Poblacional de Cáncer de Cali (RPCC) está en operación continua desde 1962 con el objetivo de producir estadísticas válidas sobre la incidencia de cáncer, sus patrones, tendencias y supervivencia.

**Métodos:**

Durante el periodo 2008-2012, se registraron 23,046 casos nuevos y durante 2011-2015 se registraron 12,761 defunciones por cáncer. La tendencia de las tasas se describió con el porcentaje de cambio medio anual APC y con el análisis de *Joinpoint*. Se analizaron los datos individuales de 38,671 adultos (15-99 años) con diagnóstico de cáncer entre 1995-2009, y se calculó la supervivencia neta estandarizada por edad para las 14 localizaciones más comunes de cáncer, con el método de Pohar-Perme.

**Resultados:**

Próstata y mama fueron la primera causa de morbilidad por cáncer. Las tasas de incidencia en estos tumores susceptibles de detección temprana se estabilizaron tras décadas de crecimiento, mientras que se observó un incremento de la incidencia de cáncer de colon y carcinoma papilar de tiroides. Las tasas de incidencia de cáncer de cuello uterino y estómago, afecciones relacionados con agentes infecciosos, disminuyeron, aunque el número de los casos absolutos aumentó, debido al crecimiento y envejecimiento de la población. El cáncer gástrico fue responsable del mayor número de muertes por cáncer. Los tipos de cáncer relacionados con el consumo de tabaco (pulmón, cavidad oral, esófago, páncreas, vejiga urinaria) mostraron cifras bajas y con tendencia al descenso. Durante el periodo 2000-2004, la supervivencia neta a 5 años mejoró para los cánceres de mama, cuello uterino, próstata, melanoma y tiroides, aunque en el periodo 2005-2009 se observó un estancamiento. En cáncer de estómago, hígado y pulmón, la supervivencia neta a 5 años fue inferior al 15%. La supervivencia global a 5 años en niños fue de 51.0% (IC 95%: 47.5, 54.3) y en adolescentes de 44.6% (IC 95%: 36.0, 52.8).

**Comentario:**

El RPCC ha sido asesor del gobierno colombiano en la evaluación de RPCs en el país y sus datos han contribuido significativamente a diferentes aspectos del control del cáncer en Colombia.

## Introduction

Comprehensive cancer control is a strategic approach that brings together the main associations and organizations of a community to prevent or minimize its impact and to develop a plan to reduce the number of citizens who become ill or die from cancer. The plans are specific, based on an analysis of the cancer situation in each country ^1^. It is essential that the information on incidence, mortality and survival be of high quality because it will help monitor and evaluate the programs. Population-based cancer registries (RCPobs) represent the gold standard for providing cancer incidence and survival figures in a region and are a key element in cancer control because they provide indicators for planning and evaluating cancer control activities and carrying out cancer research ^2^. The information disclosed by these information systems in Colombia indicates that cancer is a public health problem that causes 63,000 new cases and 33,000 deaths each year ^3^. To face this threat, the Colombian government has formulated a Ten-Year Plan for Cancer Control in Colombia (PDCC) ^4^ focusing on activities to control and reduce mortality from cancer of cervix, stomach, prostate, breast, colon and acute pediatric leukemias.

Colombia lacks an RCPobs with national coverage and for several decades the only source of valid cancer incidence information for the country was the Cali Population Registry of Cali (RPCC) ^5^. Now it has three additional regional RCPobs that provide quality cancer incidence information in Pasto ^6^, Manizales ^7^ and Bucaramanga ^8^; and two new ones in the process of consolidation in Barranquilla ^9^ and Medellín ^10^. The coverage of these six regional RCBPs is less than 12.9% of the Colombian population. To overcome this limitation, health authorities use GLOBOCAN methods to make national and regional cancer incidence estimates based on mortality information ^11^. The incidence / mortality ratio of the period of interest of each regional RCPob is incorporated into a mathematical model that uses this information and the mortality observed in each department as inputs to estimate the departmental and national incidence ^3^ ^,^ ^11^. The validity of the estimates depends on the quality of the information and also on the accurate quality of the certification of general mortality and cancer in Colombia and the coverage of the certification is close to 100% ^12^.

In this article, the Cali Population Registry discloses the most recent cancer statistics in Cali, Colombia, for incidence and mortality rates standardized by age (ASR) for all cancers for the periods 2008-2012 and 2008-2015, respectively; and the 5-year net survival estimates standardized by age for the 14 most common cancer sites from 1995 to 2009. Estimating the incidence of cancer in Colombia and creating some of the baseline indicators of the current PDCC in the city is a contribution made by the Universidad del Valle to the health authorities.

## Materials and Methods

### Population and registration area

Cali is the third largest city in Colombia, capital of the Department of Valle del Cauca. According to the 2005 census and according to the projections of the DANE ^13^, the estimated population for 2010 was 2.3 million inhabitants. 52% are females, and 26.2% self-identify as belonging to the black ethnic group ^14^. The life expectancy at birth is 73.1 years for men, and 78.5 years for females ^15^. The infrastructure for cancer care includes 165 oncology functioning services ^16^, these services are in the urban area where 95% of the population resides in an area of ​​approximately 110 km^2^ that corresponds to 20% of the extension of the municipality of Cali (503 km^2^).

### Incidence and mortality information

Information on the incidence of cancer was obtained from the database of the RPCC (2008-2012) and information on general mortality was obtained in the Municipal Public Health Secretary of Cali (2006-2015). Details on the history, objectives, logistics and coverage of the RPCC have been previously described ^5^ ^,^ ^17^. This same issue of Colombian Medical describes in detail the procedures and methods for estimating incidence, mortality and survival in adults ^18^. In summary, the RPCC was established in 1962, it is a population-based cancer registry which provides continuous information on new cases of all types of cancer in permanent residents of Cali through active search and notification.

Implementation of a childhood cancer outcomes surveillance system (VIGICANCER) within Cali’s population-based cancer registry was carried-out in 2009; methodological details have been published recently ^19^ ^,^ ^20^. Briefly, children and adolescents (<19 years of age) with new diagnosis of cancer and treated in a pediatric oncology unit of the city, are registered by the system and included in an active follow-up. VIGICANCER includes both children living in the city as well as children from other Colombian municipalities and provinces but treated in Cali. Vital status, relapse, treatment abandonment, and second neoplasms are the primary outcomes.

## Results

### 1. New cases of cancer (incidence)

In the quinquennium 2008-2012, 23,046 new cases of cancer were diagnosed among the permanent residents of Cali, for an average of 4,500 cases per year; 55% (12,613) occurred in females and the sex ratio was 1:2. The incidence rates standardized by age for all cancer sites per 100,000 person-years were 204.6 in men; and 185.1 in females. In the absence of other causes of death, the cumulative risk of developing cancer before reaching the age of 75 was 23.8% and 20.5% in males and females in Cali.

Cancer incidence rates per 100,000 person-year by sex and cancer location are shown in Table 1. In men, the five primary sites of primary cancer were prostate (ASR: 59.7), stomach (ASR: 20.2), colorectal (ASR: 16.2), lung (ASR: 14.5), and lymphomas (ASR: 11.3). Together they constituted 58.8% of all new cancer cases diagnosed between 2008 and 2012. Prostate cancer accounted for 28.2% of all incident cases, (n: 2,937).

**Table 1:** Cali, Colombia. Incidence rates standardized by age (World Population) per 100,000 person-year and the annual percentage change (APC) by sex during the period 2008-2012

	Male		Female		Male		Female		Code ICD 10
Site	n	ASR	n	ASR	APC	95% IC	APC	95% IC	
Oral cavity and pharynx	279	5.4	233	3.4	-1.2*	-1.7;-0.7	-1.1*	-1.6;-0.5	C00 14
Oesophagus	90	1.7	72	1.0	-1.3*	-2.1;-0.5			C15
Stomach	1,041	20.2	769	10.7	-1.9*	-2.1;-1.7	-1.9*	-2.1;-1.6	C16
Small intestine	39	0.7	37	0.5					C17
Colon and Rectum	831	16.2	996	1.4	2.4*	2.0;2.7	1.9*	1.5;2.3	C18 20
Anus	48	1.0	126	1.9					C21
Liver	249	5.0	218	3.1	1.7*	0.9;2.4	0.4	-0.5;1.2	C22
Gallbladder	120	2.4	264	3.7	-1.3*	-1.9;-0.6	-1.8*	-2.4;-1.2	C23 24
Pancreas	222	4.4	257	3.6			0.0	-0.5;0.5	C25
Nose, sinuses, etc.	38	0.7	18	0.3					C30 31
Larynx	202	4.0	37	0.6	-1.1*	-1.6;-0.6			C32
Trachea, bronchi and lung	731	14.5	585	8.2	-0.6*	-1.1;-0.1	0.5*	0.0;1.1	C33 34. C38 39
Bone	75	1.4	84	1.3	0.8	-0.1;1.6	0.1	-0.9;1.1	C40 41
Connective tissue	135	2.6	134	2.1			0.4	-0.2;1.0	C47 49
Mesothelioma	9	0.2	7	0.1					C45
Kaposi sarcoma	92	1.6	10	0.1					C46
Skin melanoma	145	2.8	179	2.6			1.1*	0.5;1.7	C43
Other skin	28	0.5	41	0.6					C44
Breast	26	0.5	2,972	44.3			1.4*	1.1;1.6	C50
Vulva			60	0.8			-1.6*	-2.4;-0.8	C51
Vagina			44	0.7					C52
Uterus unspecified			37	0.5					C55
Uterine cervix			1,037	15.3			-3.0*	-3.2;-2.8	C53
Corpus uteri			347	5.3			0.3	-0.1;0.7	C54
Ovary			513	7.7			-0.1	-0.5;0.2	C56
Other females genital organs			22	0.4					C57 58
Penis	74	1.3							C60
Prostate	2,937	59.7			3.0*	2.5;3.5			C61
Testicle	154	2.6			1.7*	1.0;2.5			C62
Other male genital organs	8	0.1							C63
Kidney	250	5.1	209	3.2	2.8*	2.1;3.4	2.3*	1.7;2.9	C64 66
Bladder	319	6.2	121	1.6	-0.7*	-1.1;-0.3	-1.2*	-1.7;-0.6	C67
Other urinary organs	3	0.0	4	0.1					C68
Eye	48	0.9	37	0.6					C69
Central Nervous System	271	5.2	269	4.3	1.2*	0.5;1.9	2.3*	1.2;3.4	C70 72
Thyroid	173	3.2	893	13.2	0.7	-0.1;1.5	2.6*	2.1;3.1	C73
Other endocrine	25	0.5	21	0.4					C74 75
Hodgkin's disease	91	1.7	63	1.0	-1.6*	-2.2;-0.9	-0.6	-1.4;0.2	C81
Non-Hodgkin lymphoma	502	9.6	511	7,5	2.3*	1.7;2.8	2.1*	1.6;2.7	C82 85. 96
Multiple myeloma	156	3.1	142	2,1					C90
Lymphocytic leukaemia	205	4.0	211	3,6	2.1*	1.6;2.7			C91
Myeloid and monocytic leukaemia	172	3.3	173	2,6	0.0	-0.6;0.7	1.1*	0.5;1.6	C92 94
Non-specific leukaemia	53	1.0	53	0,7					C95
Unknown primary site	581	11.3	797	11,3	-0.3	-0.7;0.1	-0.8*	-1.2;-0.4	**
All the sites	10,433	204.6	12,613	185,1	0.6*	0.4;0.8	-0.1	-0.2;0.1	C00 96
All sites *	10,405	204.1	12,572	184,5	0.6*	0.4;0.8	-0.1	-0.2;0.1	C00 43.45 96

** C26.39.48.76.80 - CIE O: 998_ / 3

Number of cases (n); Standardized incidence rate by age (ASR, by its acronym in English).

APC: For its acronym in English Annual Percent Change. APC is calculated for period 1962-2012

* All sites excluding non-melanoma skin cancer

In females, the most frequent locations for cancer according to their ASR were in descending order: breast (44.3), cervix (15.3), colorectal (14.0), thyroid (13.2), and stomach (10.7). These locations together accounted for 52.9% of all new cases of cancer diagnosed during the five-year period. Breast cancer alone accounted for 23.6% of incident cases (n: 2,972).

### 2. Mortality from cancer

Table 2 shows cancer deaths that occurred in Cali in two quinquennial periods; 2006-2010 and 2011-2015. During this decade there were 122,014 deaths, (56.8% in males and 43.2% in females). Overall mortality from cancer corresponded to 19.6% (23,873 deaths) of all deaths in that period and the number of deaths from this cause was greater among females (53.0%, 12,663) than among males (47.0%, 11,219). For the analysis of cancer mortality, emphasis was placed on the results of the quinquennium 2011-2015.

**Table 2:** Cali, Colombia. Mortality rates standardized by age (World Population) per 100,000 person-year and the annual percentage change (APC) by sex during the period 2006-2015

Localización	2006-2010				2011-2015				APC				Code ICD 10
	Male		Female		Male		Female		Male		Female		
	n	ASR	n	ASR	n	ASR	n	ASR	APC	95% CI	APC	95% CI	
Mouth and oropharynx	90	1.9	82	1.2	115	2.0	104	1.3	-2.7*	(-3.5 ; -1.9)	0.0	(-1.2 ; 1.2)	C00-14
Oesophagus	91	1.9	54	0.8	95	1.7	52	0.7	-3.5*	(-4.4 ; -2.5)	-3.9*	(-5.1 ; -2.7)	C15
Stomach	801	16.6	607	9.0	807	14.4	659	8.2	-2.3*	(-2.7 ; -2.0)	-2.5*	(-2.9 ; -2.1)	C16
Colon and rectum	421	8.6	483	7.1	570	10.2	607	7.5	2.0*	(1.4 ; 2.7)	0.4	(-0.2 ; 1.1)	C18-21
Liver	305	6.4	311	4.7	317	5.7	372	4.5	-0.2	(-0.8 ; 0.5)	-1.5*	(-2.1 ; -0.8)	C22
Pancreas	180	3.8	235	3.5	252	4.5	300	3.7	-0.6	(-1.3 ; 0.2)	-1.2*	(-2 ; -0.5)	C25
Lung	714	14.8	531	8.0	799	14.4	602	7.3	-1.8*	(-2.2 ; -1.4)	-0.8*	(-1.3 ; -0.3)	C33-34
Skin melanoma	105	2.1	102	1.4	152	2.7	126	1.6	2.2*	(1.2 ; 3.2)	1.2	(-0.1 ; 2.5)	C43-44
Breast	8	0.2	904	14.1	7	0.1	1,055	13.8			0.1	(-0.4 ; 0.6)	C50
Cervix uteri			471	7.4			487	6.5			-3.9*	(-4.3 ; -3.5)	C53
Corpus uteri			115	1.7			138	1.9			-2.3*	(-3.5 ; -1.1)	C54-C55
Ovary			297	4.7			302	4.0			-0.6	(-1.5 ; 0.3)	C56
Prostate	847	16.7			1,012	17.4			-0.1	(-0.5 ; 0.4)			C61
Bladder	100	2.0	58	0.8	113	2.0	75	0.9	-1.2*	(-2.4 ; -0.1)	-2.5*	(-3.9 ; -1.1)	C67
Lymphoma	256	5.3	237	3.8	330	6.0	290	3.7	-0.8*	(-1.5 ; 0.0)	-1.1*	(-1.9 ; -0.3)	C81-C90,C96
Leukaemia	259	5.1	286	4.6	270	4.6	246	3.5	-0.6	(-1.2 ; 0.0)	-0.6	(-1.5 ; 0.3)	C91-95
Other sites	1,003	20.6	1,159	17.8	1,191	2.1	1,316	1.7	-0.7*	(-1.1 ; -0.3)	-1.2*	(-1.6 ; -0.9)	**
All sites	5,180	10.6	5,932	90.7	6,030	10.7	6,731	85.9	-0.9*	(-1.1 ; -0.6)	-1.3*	(-1.4 ; -1.1)	C00-C97

**C17, C23, C24, C26-C32, C37-C41, C45-C49, C51, C52,C57-C60, C62-C66, C68-C80, C97

Number of cases (n); Mortality rate standardized by age (ASR).

APC: Annual Percent Change. APC is calculated for the period 1984-2015.

* The APC is significantly different from zero (p <0.05).

Cancer was the third cause of death in Cali after mortality due to cardiovascular diseases (26.0%) and unintentional or intentional injuries (20.1%).

In contrast to the number of deaths, standardized cancer mortality rates for all combined locations per 100,000 person-years were higher among males (107.0) than among females (85.9). Cancer of stomach, lung, colorectal, breast and prostate were the main causes of cancer-related death, together they represent approximately half of all cancer deaths (47.9%).

Based on mortality rates standardized by age, prostate cancer (ASR: 17.4), was the leading cause of death among tumors in males in the five-year period 2011-2015, followed by cancer of stomach (ASR: 14.4), lung (ASR: 14.4), colorectal (ASR: 10.2) and lymphomas (ASR: 6.0). Breast cancer was the leading cause of death in females (ASR: 13.8), followed by cancer of stomach (ASR: 8.2), colorectal (ASR: 7.5), lung (ASR: 7.3) and cervix (ASR: 6.5).

### 3. Changes in cancer morbidity and mortality

Tables 1 and 2 show the APC that represents the average percentage of annual increase or decrease in cancer incidence and mortality rates during the periods 1962-2012 and 1984-2015, respectively. In describing the change, three well-defined patterns were detected: increased or decreased when the APC was significantly different from zero (two-tailed values ​​p <0.05); otherwise the term stable or flat was used.

The incidence rates for all cancer body sites increased in male an annual average of 0.6% (95% CI: 0.4 - 0.8) and remained stable in females. In contrast, mortality for all cancer body sites has been significantly decreasing at an annual average of 0.9% in male; (95% CI: -1.1; -0.6); and 1.3% in females, (95% CI: -1.4, -1.1).

#### Trend in cancer incidence rates (1962-2012).

The incidence of cancer decreased in both males and females in the following sites: oral cavity and pharynx, esophagus, stomach, larynx, urinary bladder and leukemia of unspecified type. The decrease was only observed in male with pancreatic cancer and with Hodgkin's disease; and in females with cervical cancer.

In contrast, increased incidence rates of colorectal cancer, melanoma, non-Hodgkin's lymphoma and lymphoid leukemia were found in both males and females; breast and thyroid cancer increased in females only; and liver, prostate and testicular cancer in males only.

In females, there was no change in the risk of morbidity due to cancer of the liver, pancreas, lung, uterine body, ovary and Hodgkin's lymphoma and in males the incidence of thyroid cancer and myeloid leukemia remained stable.

#### Trend in cancer mortality rates (1984-2015).

Mortality from cancer shows a favorable trend. There was only an increase in mortality rate from melanoma and colorectal cancer in men. In the rest of the neoplasms, there was evidence of a decrease in mortality rates for ten of the 17 main body locations. The decrease was observed in both males and females with cancer of the esophagus, stomach, lung, urinary bladder, lymphomas and multiple myeloma; only in males with cancer of the oral cavity and pharynx; and only in females with cancer of liver, pancreas, cervix and uterine body.

There were no changes in leukemia mortality in the entire population of Cali. Mortality rates for liver, pancreas, and prostate cancer remained stable in males; and females, there were no changes in mortality rates for breast, colorectal, ovarian and melanoma cancer.

### 4. Five-year net survival

**Figure 1:**
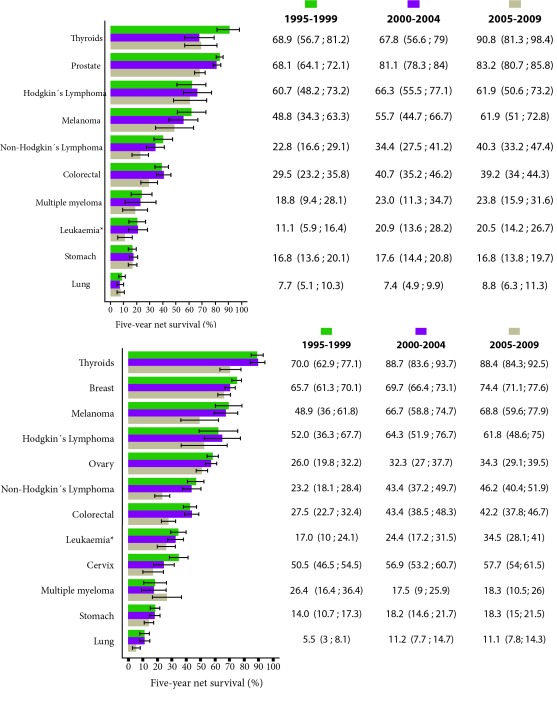
Cali, Colombia. Net standardized survival by age at 5 years for the most frequent locations by period of interest and sex, between 1995 and 2009.

For the analysis, a total of 38,671 patients diagnosed with cancer were included through 1995-2009. The distribution of the most frequent malignancies corresponded to breast (17.7%), prostate (17.3%), stomach (13.1%) and colorectal cancer (9.4%), while a smaller number of records were reported for liver cancer (2.2%), melanoma (1.8%), multiple myeloma (1.5%) and Hodgkin's lymphoma (1.0%). The median age at diagnosis for the period considered was 64 years. There has been an increase in the number of patients diagnosed for the last study period 2005-2009. The trend of net survival for certain types of cancer by sex and diagnosis period 1995-2009 is shown in Figure 1.

**Figure 2:**
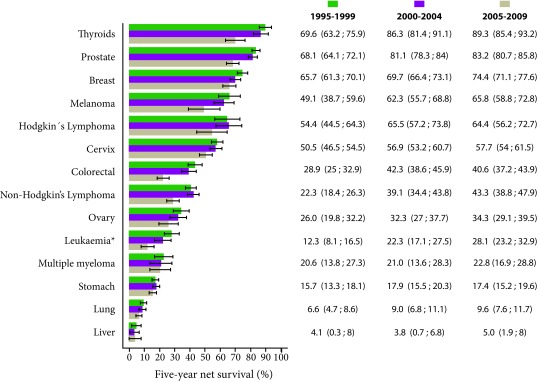
Cali, Colombia. Five-year net survival standardized by age after diagnosis by period of interest, both sexes between 1995 and 2009.

Figure 2 shows the 5-year net survival standardized by age for three quinquennial periods: 1995-1999, 2000-2004 and 2005-2009. When compared with previous periods, patients diagnosed with cancer in the most recent period (2005 -2009) marked improvements in net survival of 5 years were observed for most cancer sites. The proportions of increased cancer survival in females could be explained in part by common types of cancer in females (e.g. thyroid, breast, and cervical cancers) that have a relatively good prognosis. When examined by year of diagnosis and localization of cancer, in general terms it was evident that in the last period which includes the years 2005-2009 there was an increase in survival for most of the cancer locations except for stomach cancer and colorectal cancer.

In the case of liver cancer, age standardization was not carried out, because some of the age-specific 5-year net survival estimates necessary to carry out standardization were not available (very low survival, first year of follow-up, about 90% of patients with liver cancer died).

On the other hand, the highest estimates of net survival for the period 2005-2009 were seen for thyroid cancer (89.3%), prostate (83.2%), breast (74.4%) and melanoma (65.8%). In the case of hematolymphoid neoplasms, survival was better in patients with Hodgkin lymphoma (64.4%) than in non-Hodgkin lymphoma (43.3%). In leukemia and multiple myeloma survival was lower, with estimates for the 2005-2009 period of 28.1% and 22.8% respectively.

### Childhood cancer

VIGICANCER registered 1,428 children (<15 years of age) and adolescents (15 to 18.9 years of age) between 2010 and 2016. Ninety-six percent (n: 1,379) contributed to the follow-up (673 hemato-lymphoid tumors y 706 solid tumors). Children 5-year overall survival (OS) was 52.0% (95% CI: 48.7, 55.3) and 44.0% (95% CI: 35.4, 52.2) in adolescents. 

Table 3 shows OS by the International Childhood Cancer Classification 3rd version ^21^ cancer group. Group I was the most frequent both in children (39.7%) and adolescents (30.3%). Within this group 79.1% were acute lymphoblastic leukemia (ALL). Among group II, 38.4% were Hodgkin disease, 38.3% non-Hodgkin lymphoma (without Burkitt) and 23.3% Burkitt. From all groups, 17.2% were central nervous system tumors (group III); being the most frequent (26.5%) in the <1 year of age group.

Infancy and early childhood malignant solid tumors frequency was 2.3% for neuroblastoma (and other group IV tumors), 4.2% for retinoblastoma (group V), 3.9% for Wilms tumor (and other groups VI tumors), and 1.3% hepatoblastoma (group VII). 

Malignant bone tumors (group VIII) were more frequent in adolescents (14.9%) than in children (5.6%), with 58.9% osteosarcomas and 32.7% Ewing sarcoma. Group IX (soft tissue sarcomas) was similar in children and adolescents (5.0% vs. 5.9%). Germ cell tumors (group X) showed an overall frequency of 5.2%; in children 3.4% and in adolescents 9.4%. Epithelial malignant tumors (group XI) had higher frequency in adolescents (12.9%) than in children (2.4%). In this group, thyroid tumor was the most frequent 51.5%. Non-specified cancers (group XII) were 1.3%.

**Table 3:** Cali, Colombia, 2010-2015. Frequency of children and adolescent cancer cases and 5-year overall survival by ICCC-3 groups ^21^

Group ICCC-3	Children	%	Adolescents	%	OS	95% CI	
**I**	453	39.7	87	30.3	48.3	43.3	53.2
**II**	112	9.8	34	11.9	74.1	65.5	81.0
**III**	209	18.3	36	12.6	40.2	32.4	47.7
**IV**	32	2.8	1	0.3	39.2	21.5	56.6
**V**	60	5.3	0	0.0	74.8	61.1	84.2
**VI**	56	4.9	0	0.0	62.5	47.3	74.5
**VII**	19	1.7	0	0.0	47.8	9.6	79.3
**VIII**	64	5.6	43	15.0	29.4	18.9	40.5
**IX**	57	5.0	17	5.9	32.3	19.9	45.3
**X**	39	3.4	27	9.4	61.2	45.2	73.8
**XI**	27	2.4	37	12.9	76.2	59.8	86.6
**XII**	13	1.1	5	1.7	56.3	20.9	80.9
**Total**	1,141	100.0	287	100.0	50.8	47.7	53.8

ICCC-3: International Classification of Childhood Cancer version 3

OS: 5-year overall survival. 95% CI: 95% Confidence interval

## Discussion

The RPCC of Universidad del Valle provides unique information of the statistics of cancer in Cali, during the 2008-2015 period. This information is necessary for health authorities to make estimates of cancer risk for other regions of Colombia that are lacking cancer registries. These statistics complement previous reports ^5^ ^,^ ^22^ and provides uninterrupted continuous monitoring for the last 55 years, which allows detailed analyses of the 50 year-incidence (1962-2012), 30 year-mortality (1984-2015) and 15-year-survival (1995-2009) of cancer in the region.

Cali has experienced profound epidemiological and demographic changes in the last half of the century. The population has quadrupled and has aged, and the life expectancy at birth increased from 56.7 to 68.4 years ^13^ ^,^ ^15^; Currently there are 33 persons 65 years old or over per 100 persons under 15 (Ageing index) ^13^.

The offer of oncology care services in Cali corresponds to one sixth of the country's installed capacity ^16^ and attends around 9,000 new cases of cancer per year, half are permanent residents and the rest are patients from the south-west, a region that represents 20% of the Colombian population ^13^. Eighty five percent of the oncology services in Cali are private ^16^, the care is not comprehensive and there are several barriers to accessing quality oncological care services. Government measures aimed at stabilizing the health system have been unsuccessful and there has been evidence of discriminatory behavior and risk selection of the oncological patients by the health care provider entities responsible for managing the risks related to the disease ^23^. Therefore, the clinical outcomes remain unfavorable primarily because patients present late with in advanced stages of the disease and, thus, survival is low for most types of cancer compared to that observed in Europe and the United States ^24^ ^,^ ^25^.

Coinciding with demographic changes there are significant variations in trends, patterns and differences in incidence rates and cancer mortality. The increase or decrease in the risk of morbidity and mortality due to this group of diseases is determined by different factors. So far, some are recognized and most are still to be identified. These changes may be the result of variations in the exposure of the population to different risk factors, better access to health services and improvement in diagnostic and treatment techniques ^26^ ^,^ ^27^.

Although several threats persist, the available information shows evidence of advances in the control of some types of cancer in Cali. Overall cancer mortality decreased significantly in both males and females with an annual change rate of 1% during the period 1984-2015 ((APC: -0.9, 95% CI: -1.1; -0.6) and (APC: -1.3, 95% CI: -1.4; -1.1)). The magnitude of the decrease was greater in patients with cancer related to tobacco consumption, infectious agents and hematolymphoid neoplasms where important therapeutic advances have been made (Fig. 3).

**Figure:**
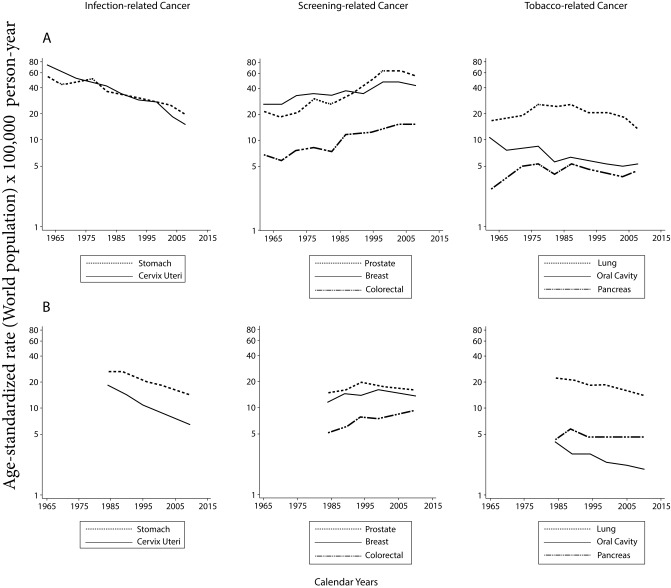

**Figure 3.** Cali, Colombia. Trend in cancer rates in the last 55 years

### Cancer related to infectious agents

The incidence and mortality rates for stomach and cervix uteri cancer have decreased significantly over the last 55 years (Fig. 3). The descent is monotonic, continuous and began before knowing the prominent role in the processes of carcinogenesis of *Helicobacter pylori* ^28^ and the Human Papilloma Virus (HPV) ^29^. These changes are not related to specific interventions against these infectious agents, they are the result of progress in the development and improvement of sanitary conditions. Economic development determined changes in lifestyles and modifications of the known risk factors for gastric cancer. Refrigeration facilitates the consumption of fresh foods and limits the use of chemical-based food preservation methods (salting, desiccation, smoking, and acidification).

In the 21st century, gastric cancer still represents a great social burden in Cali and Colombia because it causes the highest number of deaths from cancer ^3^ ^,^ ^5^ ^,^ ^22^. The disease is fatal when discovered clinically because the diagnosis is usually made in the advanced stages. From 1995 to 2009, the 5-year net survival of patients with gastric cancer in Cali was less than 20%, with a healing fraction of 15% and an average survival time of 6 months for uncured cases ^22^; similar results were observed in Chile and Costa Rica. But in other Latin American countries with equal or lesser development, the survival estimates were around 30%, which are comparable to those observed in the United States and Europe. It is also possible that estimates of gastric cancer survival in Ecuador and Cuba may be overestimated ^24^ ^,^ ^25^.

Despite the continuous decline in the incidence and mortality of infection-related cancers, rates remain high and the number of cases continues to increase due to aging and population growth ^30^. It is very likely that this downward trend will continue, even without additional interventions, in the years to come; however, under natural conditions, it will likely take many decades, if not centuries, before the incidence and mortality rates reach the values ​​currently observed in the United States and Europe. It is a priority to implement additional measures to accelerate the decline, improve survival and achieve control ^30^.

The perspectives for the control of gastric cancer are uncertain because therapeutic advances are insufficient, the pre-clinical results of efforts to develop vaccines against *H. pylori* have been disappointing ^31^; and the early detection of gastric cancer in Latin America has shown unreliable results ^32^ ^,^ ^33^ and low cost-effectiveness ^34^. During the first stages of tumor growth, cancer is clinically silent. Therefore, an alternative to control, is the implementation of primary prevention programs which would help eradicate *H. pylori* infection by reducing the risk of developing gastric cancer in people without precursor lesions ^30^ ^,^ ^34^ ^,^ ^35^. Due to the above, it is necessary to develop 1) monotherapies to facilitate adherence to antibiotic treatment and 2) accurate non-invasive tests to identify premalignant gastric lesions and thus serve as a risk stratification tool of patients. The simultaneous detection of serum pepsinogens and antibodies against *H. pylori* has achieved this goal in Japan ^35^ ^,^ ^36^. This strategy has not been adequately validated in Latin America and continues to be an option that requires exploration with a well-founded project of implementation.

The picture is different and more favorable for females with cervix uteri cancer (CUC). Mortality rates in Cali are close to the PDPCC goal ^4^, but they are still three times higher compared to the United States and Europe; where the risk of cervix uteri cancer is half of that observed in Cali. The incidence and mortality rates have declined for many reasons, including declining fertility rates, improved socio-economic conditions and the establishment of a citywide program to prevent cervix uteri cancer via a widespread use of pap smear ^29^ ^,^ ^37^.

The knowledge that certain genotypes of VPH infection are necessary to cause cervix uteri cancer has created new strategies for its prevention in the current PDPCC. As of 2012, the national guidelines for vaccination against VPH are established and coverage of 80% has been achieved, which unfortunately is now below 10% because of a mismanaged episode of massive psychogenic reaction in Carmen de Bolívar, a Colombian rural region. This reaction was supposedly associated with the VPH vaccine ^38^. To increase the accuracy of cervix uteri screening, the Ministry of Health of Colombia incorporated HPV testing in cervical cancer screening programs. It is expected to achieve coverage of 80% of the target population in 2021 ^4^. These measures are essential to accelerate the control of this disease because the 5-year net survival of females with cervix uteri cancer in Cali was 57%, 10% points below that observed in affluent countries ^24^ ^,^ ^25^.

### Cancer related opportunity screening activities

Prostate and breast cancer are the leading cause of cancer-related morbidity in males and females in Cali, respectively ^5^. In Colombia there are no organized screening programs for either cancer and cancer control is based on specific opportunistic screening activities. Mammography, digital rectal examination and PSA allowed us to detect cases of disease that were previously unknown and contributed to increasing the incidence rates before the first quinquennium of the 21st century and since then, it has begun to decline. Most, but not all, of the increase may be due to earlier detection of the disease. Once the use of screening tests had been established the rates tended to stabilize as long as other factors causing the disease had not changed. 

These changes were more evident in the population subject to screening, the group of 50-69 years of age, where there was also a turning point in the trend of incidence. Similar changes were documented in Costa Rica and Ecuador at the end of the first decade of the 21st century and were observed in Europe and the United States 20 years earlier.

Mortality from prostate cancer has decreased consistently since 1984 with an average annual percentage decrease of 2%; the decline occurred earlier than expected. This could not be attributed exclusively to the screening activities (Fig. 3). An influential and perhaps determining factor is the evolution of treatments with curative intent; it is likely that the use of PSA and digital rectal examination have contributed to maintaining and consolidating this trend ^39^. However, mortality from breast cancer remained stable during the study period (Fig. 3).

The United States and Europe have made great advances in the control of prostate and breast cancer. Despite the high incidence rates (ASR: 119.8 and ASR: 124.9, respectively); the 5-year net survival is around 98.9% and 89.7%; and mortality rates around (ASR: 20.1, ASR: 21.2), respectively ^11^ ^,^ ^24^. In Cali, 5-year net survival for the same neoplasms was 83.2% and 74.4%; and the mortality rates around (ASR: 17.4 and ASR: 13.8), respectively. The existence of a gap of 15 percentage points in 5-year net survival in a population where incidence rates are half of those observed in affluent countries ^40^, suggests that the diagnosis of cases is made at more advanced stages and / or that the tumors are more aggressive. This will remain an area of future investigation.

The incidence and mortality due to colorectal cancer continues to rise in males and females in Cali. The reasons are that the screening activities are incipient, and the risk factors are difficult to control or are not clearly identified ^41^; it is a priority to promote an organized screening program to reverse the current trend. Until this intervention occurs, oncological care services must be oriented to the early diagnosis of suspected cases.

### Cancer related to tobacco use

The trend in the incidence of lung cancer correlates with the historical patterns of prevalence of cigarette smoking and there is sufficient evidence of a causal relationship between cigarette smoking and various types of cancer. The reduction in the number of cancer cases related to tobacco use in Cali, has been interpreted as a successful example of cancer control. This was due to the implementation of a very strong anti-smoking government campaign implemented in the seventies and that has been consolidated in 

the 21st century. The incidence rates of lung cancer for both sexes in Cali reflect the end of a tobacco-related epidemic that began in the 1970s and was interrupted around the 1980s ^5^ ^,^ ^42^ ^,^ ^43^. Since then, there has been a significant decrease in tobacco-related cancer incidence and mortality: oral cavity and pharynx, esophagus, pancreas, lung and urinary bladder. The decrease was more consistent in the oral cavity and lung cancer in both males and females.

### Net survival estimates

Surveillance of cancer survival is important for health organizations, civil society and research agencies because it serves to formulate strategies and prioritize cancer control measures, and to evaluate effectiveness, as well as the cost effectiveness of these strategies ^1^.

At the beginning of the 21st century, we began to monitor trends in cancer survival in Cali. The relative survival (without age-adjustment) was estimated for 16,064 patients diagnosed with prostate, breast, colorectal, cervical, stomach and lung cancer through 1995-2004 ^22^. The present study, covers 38,671 patients diagnosed with invasive primary cancer in 14 body locations representing around 71.8% of the global cancer burden in Cali (15-year period 1995-2009). Furthermore, the accuracy of the previous estimates was improved through the implementation of the new unbiased Pohar-Perme estimator ^44^ ^-^ ^46^.

Coinciding with the implementation of the new health system in the 1990s, survival improved for most of the neoplasms in the first five-year period of the 21st century compared to the 1995-1999 period. This trend stagnated in the five-year period 2005-2009. The 5-year net survival was like that found in Argentina, Chile, Ecuador and Costa Rica and very low compared to that observed in developed countries ^24^.

### Certification of cancer mortality

Information on cancer mortality in liver, lung, brain and bones should be interpreted with caution, because in these sites, the occurrence of metastasis is frequent. In the Cali cancer registry, we found evidence that the primary site of some of these cancers came from locations different than these organs. It is important to understand that 45% of liver cancer cases corresponded to metastasis. It was also established that cancers of the bone (46%), lung (15%) and CNS (10%) corresponded to metastasis. There were 2,447 new cases and 450 deaths from cancer. The coding of the body locations made by the vital statistics office and the cancer registry were compared. The concordance (Landis criteria ^47^ for the coding of cases of liver, bone and lung cancer were considerable (Kappa = 0.64, 0.67 and 0.79, respectively) and the highest was for malignant tumors of the CNS (Kappa = 0.90). 

### Childhood cancer

About 200,000 new childhood cancer cases per year are diagnosed in the world ^48^, 84% occurring in low and middle-income countries ^49^ ^,^ ^50^. Taken into account that cancer in children is not amenable to primary or secondary prevention, survival is the most relevant metric to evaluate efforts aimed to control cancer burden in this population group. Cali’s 5-year OS (51%) is 26% to 32% lower to outcomes reported in more affluent countries (77% to 83%) ^51^ ^,^ ^52^. This implies that if in Colombia 1,500 to 1,600 children are treated for cancer per year then after 5 years 765 to 816 had died, and 390 to 512 would be preventable deaths. This survival gap persists in all cancer groups, except for Hodgkin disease (7% difference; 5-year OS: 88% vs 95%). Effectiveness decrease in cancer treatment is mainly related to intensity lost. Chemotherapy intensity is related both to dose and time interval among doses. Therefore, effectiveness of treatment is very dependent on the delays in treatment administration (adherence to treatment), being treatment abandonment the extreme example of this principle. Intensity lost has multifactorial causes involving the patient, their families, health providers, and the health system. Other path to reduce survival is due to treatment mortality and not because disease. This adverse outcome is both related to access to supportive care and advance disease at diagnosis. Access to timely and correct diagnosis and treatment is particularly related to poor outcomes in tumors that are dependent of the stage at diagnosis to achieve cure; retinoblastoma is the best example of this. Nevertheless, in the Latin-American context, the Argentinian hospital registries system reports a 3-year OS of 61.7% ^53^, which compares favorably with Cali´s 3-year OS of 56.6% for this cohort.

### Limitations 

RPCC does not actively monitor adults, and Cali lacks reliable statistics on population migration ^13^. The RPCC has information about the cause of death through death certificates, but in some cases it can be difficult to determine if cancer is the basic cause of death.

### Strengths

The RPCC has participated in many other collaborative studies and has been an advisor to the Colombian government in the evaluation of PBCR in the country and its data have contributed significantly to different aspects of cancer control in Colombia. The collaborative work with the SSPM of Cali facilitates access to information on general mortality and cancer; and allows an independent source of verification of new cases of cancer. Access to the information system of the Ministry of Health (SISPRO) and to the assurance databases provides a permanent update of the vital status and date of last contact.

## References

[1] Townsend JS, Moore AR, Mulder TN, Boyd M (2015). What does a Performance Measurement System Tell Us about the National Comprehensive Cancer Control Program. J Publ Health managem Practice.

[2] Bray F, Znaor A, Cueva P, Korir A, Swaminathan R, Ullrich A (2015). Planning and developing population-based cancer registration in low- and middle-income settings.

[3] Pardo C, Cendales R (2018). Cancer incidence estimates and mortality for the top five cancer in Colombia 2007-2011. Colomb Med (Cali).

[4] Ministerio de Salud y Protección Social.Instituto Nacional de Cancerología (2012). Plan Decenal para el Control del Cáncer en Colombia, 2012 - 2021.

[5] Bravo LE, Collazos T, Collazos P, García LS, Correa P (2012). Trends of cancer incidence and mortality in Cali, Colombia. 50 years experience. Colomb Med (Cali).

[6] Yepez MC, Jurado DM, Bravo LM, Bravo LE (2018). Trends in cancer incidence, and mortality in Pasto, Colombia. 15 years experience. Colomb Med (Cali).

[7] Arias-Ortiz N, López-Guarnizo G, Arboleda-Ruiz W (2012). Cancer incidence and mortality in Manizales 2003-2007. Colomb Med (Cali).

[8] Uribe PCJ, Serrano GSE, Hormiga SCM (2018). Cancer incidence and mortality in Bucaramanga, Colombia. 2008-2012. Colomb Med (Cali).

[9] Vargas Moranth R, Navarro Lechuga E (2018). Cancer incidence and mortality in Barranquilla, 2008-2012. Colomb Med (Cali).

[10] Brome BMR, Montoya RDM, Salcedo LA (2018). Cancer incidence and mortality in Medellin-Colombia, 2010-2014. Colomb Med (Cali).

[11] Ferlay J, Soerjomataram I, Dikshit R, Eser S, Mathers C, Rebelo M (2015). Cancer incidence and mortality worldwide sources, methods and major patterns in GLOBOCAN 2012. Int J Cancer.

[12] Cendales R, Pardo C (2018). Quality of death certification in Colombia. Colomb Med (Cali).

[13] DANE (2010). Estimaciones y proyecciones de población periodo 1985-2020.

[14] DANE (2011). Cuantos somos Como vamos. Diagnóstico Sociodemográfico de Cali y 10 municipios del Pacífico nariñense. Departamento Administrativo Nacional de Estadística DANE - Equipo de Grupos Étnicos de la Dirección de Censos y Demografía.

[15] Pan American Health Organization (2012). Health in the Americas: 2012 Edition. Regional Outlook and Country Profiles.

[16] Suarez MA, Aguilera J, Salguero EA, Wiesner C (2018). Pediatric oncology services in Colombia. Colomb Med (Cali).

[17] Correa P, Llanos G (1966). Morbidity and mortality from cancer in Cali, Colombia. J Natl Cancer Inst.

[18] García LS, Bravo LE, Collazos P, Ramírez O, Carrascal E, Nuñez M, Portilla N, Millán E (2018). Methods and procedures of the Population-Based Cancer Registry of Cali, Colombia. Colomb Med (Cali).

[19] Ramirez O, Aristizabal P, Zaidi A, Ribeiro RC, Bravo LE, VIGICANCER Working Group (2018). Implementing a childhood cancer outcomes surveillance system within a population-based cancer registry. J Global Oncol.

[20] Bravo LE, García LS, Collazos P, Aristizabal P, Ramirez O (2013). Descriptive epidemiology of childhood cancer in Cali, Colombia 1977-2011. Colomb Med (Cali).

[21] Steliarova-Foucher E, Stiller C, Lacour B, Kaatsch P (2005). International Classification of Childhood Cancer. Cancer.

[22] Bravo LE, García LS, Collazos PA (2014). Cancer survival in Cali, Colombia A population-based study, 1995-2004. Colomb Med (Cali).

[23] Sanz C (2017). Out-of-Sync Cancer Care Health Insurance Companies, Biomedical Practices, and Clinical Time in Colombia. Med Anthropol.

[24] Allemani C, Weir HK, Carreira H, Harewood R, Spika D, Wang XS (2015). Global surveillance of cancer survival 1995-2009: analysis of individual data for 25,676,887 patients from 279 population-based registries in 67 countries (CONCORD-2). Lancet.

[25] Allemani C, Matsuda T, Di Carlo V, Harewood R, Matz M, Nikšić M (2018). Global surveillance of trends in cancer survival 2000-14 (CONCORD-3): analysis of individual records for 37 513 025 patients diagnosed with one of 18 cancers from 322 population-based registries in 71 countries. Lancet.

[26] Gelband H, Sloan FA (2007). Cancer control opportunities in low-and middle-income countries.

[27] Torre LA, Siegel RL, Ward EM, Jemal A (2016). Global cancer incidence and mortality rates and trends-an update. Cancer Epidemiol Biomarkers Prev.

[28] Piazuelo MB, Correa P (2013). Gastric cancer overview. Colomb Med (Cali).

[29] Muñoz N, Bravo LE (2012). Epidemiology of cervical cancer in Colombia. Colomb Med (Cali).

[30] IARC, Helicobacter pylori Working Group (2014). *Helicobacter pylori* Eradication as a strategy for preventing gastric cancer.

[31] Rugge M, Genta RM, Di Mario F, El-Omar EM, El-Serag HB, Fassan M (2017). Gastric cancer as preventable disease. Clin Gastroenterol Hepatol.

[32] Pisani P, Oliver WE, Parkin DM, Alvarez N, Vivas J (1994). Case-control study of gastric cancer screening in Venezuela. Br J Cancer.

[33] Rosero-Bixby L, Sierra R (2007). X-ray screening seems to reduce gastric cancer mortality by half in a community-controlled trial in Costa Rica. Br J Cancer.

[34] Llorens P (1991). Gastric cancer mass survey in Chile. Semin Surg Oncol.

[35] Sugano K (2007). Prevention of gastric cancer urgent need to implement Helicobacter pylori eradication therapy as a primary preventive measure in Japan. J Gastroenterol.

[36] Sugano K (2016). Strategies for Prevention of Gastric Cancer Progress from Mass Eradication Trials. Dig Dis.

[37] Aristizabal N, Cuello C, Correa P, Collazos T, Haenszel W (1984). The impact of vaginal cytology on cervical cancer risks in Cali, Colombia. Int J Cancer.

[38] Palacios R (2014). Considerations on immunization anxiety-related reactions in clusters. Colomb Med (Cali).

[39] Restrepo J A, Bravo LE, García-Perdomo HA, García LS, Collazos P, Carbonell J (2014). Incidencia, mortalidad y supervivencia al cáncer de próstata en Cali, Colombia, 1962-2011. Salud Pública Mex.

[40] Ferlay J, Soerjomataram I, Ervik M, Dikshit R, Eser S, Mathers C GLOBOCAN 2012: estimated cancer incidence, mortality and prevalence worldwide in 2012.

[41] Cortes A, Bravo LE, García LS, Collazos P (2014). Incidencia, mortalidad y supervivencia por cáncer colorrectal en Cali, Colombia, 1962-2012. Salud Pública Mex.

[42] Bosetti C, Malvezzi M, Chatenoud L, Negri E, Levi F, La Vecchia C (2005). Trends in cancer mortality in the Americas, 1970-2000. Ann Oncol.

[43] Menezes A, Lopez M, Hallal P, Muiño A, Perez-Padilla R, Jardim J (2009). Prevalence of smoking and incidence of initiation in the Latin American adult population the PLATINO study. BMC Pub Health.

[44] Perme M, Stare J, Estève J (2012). On estimation in relative survival. Biometrics.

[45] Clerc-Urmès I, Grzebyk M, Hédelin G (2014). Net survival estimation with stns. Stata J.

[46] Danieli C, Remontet L, Bossard N, Roche L, Belot A (2012). Estimating net survival the importance of allowing for informative censoring. Stat Med.

[47] Landis JR, Koch GG (1977). The measurement of observer agreement for categorical data. Biometrics.

[48] Kellie SJ, Howard SC (2008). Global child health priorities: What role for paediatric oncologists?. Eur J Cancer.

[49] Rodriguez-Galindo C, Friedrich P, Alcasabas P, Antillon F, Banavali S, Castillo L (2015). Toward the cure of all children with cancer through collaborative efforts pediatric oncology as a global challenge. J Clin Oncol.

[50] Magrath I, Steliarova-Foucher E, Epelman S, Ribeiro RC, Harif M, Li CK (2013). Paediatric cancer in low-income and middle-income countries. Lancet Oncol.

[51] Gatta G, Botta L, Rossi S, Aareleid T, Bielska-Lasota M, Clavel J (2014). Childhood cancer survival in Europe 1999-2007: results of EUROCARE-5-a population-based study. Lancet Oncol.

[52] Ward E, DeSantis C, Robbins A, Kohler B, Jemal A (2014). Childhood and adolescent cancer statistics. 2014. CA Cancer J Clin.

[53] Moreno F, Dussel V, Orellana L (2015). Childhood cancer in Argentina Survival 2000-2007. Cancer Epidemiol.

