# Supplementary material for: Reliable information for cancer control in Cali, Colombia
Source: Colomb Med (Cali). 2018 Mar 30;49(1):23–34. doi: 10.25100/cm.v49i1.3689 (PMC6018818; doi:10.25100/cm.v49i1.3689)
Supplement: Related texts

# Research article: Información fiable para el control del cáncer en Cali, Colombia

## Introducción

El control integral del cáncer es un enfoque estratégico que reúne a las principales asociaciones y organizaciones de una comunidad para prevenir o minimizar su impacto y para desarrollar un plan para reducir el número ciudadanos que enferman o mueren por cáncer. Los planes son específicos con base en un análisis de la situación de cáncer de cada país ^1^. Es fundamental que la información de incidencia, mortalidad y supervivencia sea de calidad porque con ella se hará el seguimiento y la evaluación de los programas. Los registros de cáncer con base en la población (RCPobs) representan el estándar de oro para suministrar las cifras de incidencia y supervivencia al cáncer en una región y son un elemento clave para el control del cáncer porque proporcionan indicadores para planificar y evaluar las actividades de control del cáncer y realizar investigaciones ^2^. La información divulgada por estos sistemas de información en Colombia, indican que el cáncer es un problema de salud pública que ocasiona cada año 63,000 casos nuevos y 33,000 defunciones ^3^. Para enfrentar esta amenaza, el gobierno colombiano formula el Plan Decenal para el Control del Cáncer en Colombia (PDCC) ^4^ y centra sus actividades en el control del cáncer de cuello uterino, estómago, próstata, mama, colon y leucemia aguda pediátrica; para disminuir la mortalidad por este grupo de enfermedades. 

Colombia carece de un RCPob con cobertura nacional y durante varias décadas la única fuente de información válida de incidencia de cáncer para el país fue el Registro Poblacional de Cáncer de Cali (RPCC) ^5^. Ahora cuenta con tres RCPobs regionales adicionales que proporcionan información de incidencia de cáncer de calidad en Pasto ^6^, Manizales ^7^ y Bucaramanga ^8^; y dos nuevos en proceso de consolidación en Barranquilla ^9^ y Medellín ^10^ La cobertura de estos seis RCBPs regionales es inferior al 12.9% de la población colombiana. Para superar esta limitación las autoridades sanitarias utilizan los métodos de GLOBOCAN para hacer las estimaciones de la incidencia de cáncer nacional y regional con base en la información de la mortalidad ^11^. La razón incidencia/mortalidad del periodo de interés de cada RCPob regional, se incorpora en un modelo matemático que utiliza esta información y la mortalidad observada en cada departamento como insumos para estimar la incidencia departamental y nacional ^3^^,^^11^. La validez de las estimaciones depende de la calidad de la información, y la calidad de la certificación de la mortalidad general y por cáncer en Colombia es buena y la cobertura de la certificación es cercana al 100% ^12^.

En este artículo, el Registro Poblacional de Cáncer de Cali divulga las estadísticas más recientes del cáncer en Cali, Colombia, incluyendo las tasas de incidencia y mortalidad estandarizadas por edad (ASR por sus siglas en inglés) para todo el cáncer a través de los periodos 2008-2012 y 2008-2015, respectivamente; y las estimaciones de supervivencia neta a 5 años estandarizadas por edad, para las 14 localizaciones más comunes de cáncer desde 1995 a 2009. Es un aporte de la Universidad del Valle a las autoridades sanitarias para realizar la estimación de la incidencia por cáncer en Colombia y para la construcción de algunos de los indicadores de la línea de base del actual PDCC en la ciudad. 

## Materiales y Métodos

### Población y área de registro

Cali es la tercera ciudad de Colombia, capital del Departamento del Valle del Cauca. Según el censo del 2005 y de acuerdo a las proyecciones del DANE ^13^, la población estimada para el 2010 fue 2.3 millones de habitantes, 52.0% son mujeres, y 26.2% se auto reconocen como pertenecientes a la etnia negra ^14^. La expectativa de vida al nacimiento es 73.1 años para hombres, y 78.5 años para las mujeres ^15^. La infraestructura para la atención oncológica cuenta con 165 servicios oncológicos habilitados ^16^, estos servicios se encuentran en el área urbana donde reside el 95% de la población en un área de 110 km que corresponde al 20% de la extensión del municipio de Cali (503 km^2^).

### Información de la incidencia y mortalidad

La información sobre la incidencia de cáncer se obtuvo de la base de datos del RPCC (2008-2012) y la información de la mortalidad general se consiguió en la Secretaría de Salud Pública Municipal de Cali (2006-2015). Los detalles relativos a la historia, los objetivos, la logística y la cobertura del RPCC se han descrito previamente ^5^^,^^17^. En este mismo número de la Revista Colombia Médica se describe en detalle los procedimientos y métodos para las estimaciones de incidencia, mortalidad y supervivencia en adultos ^18^. En resumen, el RPCC fue establecido en 1962, es un registro de cáncer basado en la población, el cual proporciona información continua sobre los casos nuevos de todos los tipos de cáncer, en residentes permanentes de Cali mediante búsqueda activa y notificación. 

En el 2009 se implementó dentro del RPCC un sistema de información para el monitoreo continuo de los resultados clínicos de niños con cáncer tratados en Cali (VIGICANCER), cuyos detalles ya han sido publicados ^19^^,^^20^. En resumen, el sistema además del registro de casos incidentes, hace seguimiento activo a los menores de 19 años tratados en Cali en unidades de oncología pediátrica. El sistema incluye tanto residentes de la ciudad como pacientes remitidos de otros municipios y departamentos. 

## Resultados

### 1. Casos nuevos de cáncer (incidencia)

En el quinquenio 2008-2012 se diagnosticaron 23,046 casos nuevos de cáncer entre los residentes permanentes de Cali, para una media de 4,500 casos anuales; 55% (12,613) ocurrieron en las mujeres y la razón por sexo fue de 1.2. Las tasas de incidencia estandarizadas por edad para todas las localizaciones del cáncer fueron 204.6 en hombres; y 185.1 en las mujeres. En ausencia de otras causas de muerte, el riesgo acumulado de desarrollar un cáncer antes de llegar a los 75 años fue 23.8% y 20.5% en los hombres y en las mujeres de Cali.

Las tasas de incidencia de cáncer por cada 100,000 personas-año por sexo y localización del cáncer se muestran en la Tabla 1. En los hombres, las cinco localizaciones principales de cáncer primario fueron próstata (ASR: 59.7), estómago (ASR: 20.2), colon y recto (ASR: 16.2), pulmón (ASR: 14.5), y linfomas (ASR: 11.3). En conjunto constituyeron el 58.8% de todos los casos nuevos de cáncer diagnosticados entre 2008 y 2012. El cáncer de próstata representó el 28.2% de todos los casos incidentes, (n: 2,937).

**Tabla 1:** Cali, Colombia. Tasas de incidencia estandarizadas por edad (Población Mundial) por 100,000 per-año y el cambio porcentual anual (APC, por su sigla en inglés) según sexo durante el periodo 2008-2012.

	Hombre		Mujer		Hombre		Mujer		Código ICD 10
Localización	*n*	ASR	*n*	ASR	APC	IC 95%	APC	IC 95%	
Cavidad oral y faringe	279	5.4	233	3.4	-1.2*	-1.7;-0.7	-1.1*	-1.6;-0.5	C00 14
Esófago	90	1.7	72	1.0	-1.3*	-2.1;-0.5			C15
Estómago	1.041	20.2	769	10.7	-1.9*	-2.1;-1.7	-1.9*	-2.1;-1.6	C16
Intestino delgado	39	0.7	37	0.5					C17
Colon y Recto	831	16.2	996	1.4	2.4*	2.0;2.7	1.9*	1.5;2.3	C18 20
Ano	48	1.0	126	1.9					C21
Hígado	249	5.0	218	3.1	1.7*	0.9;2.4	0.4	-0.5;1.2	C22
Vesícula biliar	120	2.4	264	3.7	-1.3*	-1.9;-0.6	-1.8*	-2.4;-1.2	C23 24
Páncreas	222	4.4	257	3.6			0.0	-0.5;0.5	C25
Nariz, senos, etc.	38	0.7	18	0.3					C30 31
Laringe	202	4.0	37	0.6	-1.1*	-1.6;-0.6			C32
Tráquea, bronquios y pulmón	731	14.5	585	8.2	-0.6*	-1.1;-0.1	0.5*	0.0;1.1	C33 34. C38 39
Hueso	75	1.4	84	1.3	0.8	-0.1;1.6	0.1	-0.9;1.1	C40 41
Tejido Conectivo	135	2.6	134	2.1			0.4	-0.2;1.0	C47 49
Mesotelioma	9	0.2	7	0.1					C45
Sarcoma de Kaposi	92	1.6	10	0.1					C46
Melanoma de piel	145	2.8	179	2.6			1.1*	0.5;1.7	C43
Otros de piel	28	0.5	41	0.6					C44
Mama	26	0.5	2,972	44.3			1.4*	1.1;1.6	C50
Vulva			60	0.8			-1.6*	-2.4;-0.8	C51
Vagina			44	0.7					C52
Útero inespecífico			37	0.5					C55
Cérvix uterino			1,037	15.3			-3.0*	-3.2;-2.8	C53
Cuerpo Uterino			347	5.3			0.3	-0.1;0.7	C54
Ovario			513	7.7			-0.1	-0.5;0.2	C56
Otros órganos genitales femeninos			22	0.4					C57 58
Pene	74	1.3							C60
Próstata	2.937	59.7			3.0*	2.5;3.5			C61
Testículo	154	2.6			1.7*	1.0;2.5			C62
Otros órganos genitales masculinos	8	0.1							C63
Riñón	250	5.1	209	3.2	2.8*	2.1;3.4	2.3*	1.7;2.9	C64 66
Vejiga	319	6.2	121	1.6	-0.7*	-1.1;-0.3	-1.2*	-1.7;-0.6	C67
Otros órganos urinarios	3	0.0	4	0.1					C68
Ojo	48	0.9	37	0.6					C69
Sistema nervioso central	271	5.2	269	4.3	1.2*	0.5;1.9	2.3*	1.2;3.4	C70 72
Tiroides	173	3.2	893	13.2	0.7	-0.1;1.5	2.6*	2.1;3.1	C73
Otros endocrinos	25	0.5	21	0.4					C74 75
Enfermedad de Hodgkin	91	1.7	63	1.0	-1.6*	-2.2;-0.9	-0.6	-1.4;0.2	C81
Linfoma no Hodgkin	502	9.6	511	7.5	2.3*	1.7;2.8	2.1*	1.6;2.7	C82 85. 96
Mieloma múltiple	156	3.1	142	2.1					C90
Leucemia linfocítica	205	4.0	211	3.6	2.1*	1.6;2.7			C91
Leucemia mieloide y monóocitica	172	3.3	173	2.6	0.0	-0.6;0.7	1.1*	0.5;1.6	C92 94
Leucemia inespecífica	53	1.0	53	0.7					C95
Sitio primario desconocido	581	11.3	797	11.3	-0.3	-0.7;0.1	-0.8*	-1.2;-0.4	**
Todos los sitios	10,433	204,6	12,613	185.1	0.6*	0.4;0.8	-0.1	-0.2;0.1	C00 96
Todos los sitios *	10,405	204.1	12,572	184.5	0.6*	0.4;0.8	-0.1	-0.2;0.1	C00 43.45 96

**C26.39.48.76.80 - CIE O:998_/3

Número de casos (n);

ASR: Tasa de incidencia estandarizada por edad (ASR, por su sigla en inglés).

APC: Por su sigla en inglés Annual Percent Change. APC es calculado para periodo 1962-2012

*Todos los sitios excluyendo cáncer de piel no melanoma

En las mujeres, las localizaciones más frecuentes para cáncer de acuerdo a sus ASR fueron en orden descendente: mama (ASR: 44.3), seguido por cuello uterino (ASR: 15.3), colon y recto (ASR: 14.0), tiroides (ASR: 13.2), y estomago (ASR: 10.7). Estas localizaciones representaron en conjunto el 52.9% de todos los casos nuevos de cáncer diagnosticados durante el quinquenio. El cáncer de mama por sí solo constituyó el 23.6% de los casos incidentes (n: 2,972).

### 2. Mortalidad por cáncer

La Tabla 2 muestra las defunciones por cáncer que ocurrieron en Cali en dos quinquenios; 2006-2010 y 2011-2015. Durante la década hubo 122,014 defunciones, (56.8% en hombres y 43.2% en mujeres). La mortalidad global por cáncer corresponde al 19.6% (23,873 defunciones) de todas las muertes de ese periodo y el número de defunciones por esta causa fue mayor entre las mujeres (53.0%, 12,663), que entre los hombres (47.0%, 11,219). Para el análisis de la mortalidad por cáncer se hará énfasis en los resultados del quinquenio 2011-2015. 

**Tabla 2:** Cali, Colombia. Tasas de mortalidad estandarizadas por edad (Población Mundial) por 100.000 per-año y el cambio porcentual anual (APC, por su sigla en inglés) según sexo durante el periodo 2006-2015

Localización	2006-2010				2011-2015				APC				Código ICD 10	
	Hombre		Mujer		Hombre		Mujer		Hombre		Mujer		
	*n*	ASR	*n*	ASR	*n*	ASR	*n*	ASR	APC	95% C.I.	APC	95% C.I.	
Boca y orofaringe	90	1.9	82	1.2	115	2.0	104	1.3	-2.7*	(-3.5 ; -1.9)	0.0	(-1.2 ; 1.2)	C00-14
Esófago	91	1.9	54	0.8	95	1.7	52	0.7	-3.5*	(-4.4 ; -2.5)	-3.9*	(-5.1 ; -2.7)	C15
Estómago	801	16.6	607	9.0	807	14.4	659	8.2	-2.3*	(-2.7 ; -2.0)	-2.5*	(-2.9 ; -2.1)	C16
Colon y recto	421	8.6	483	7.1	570	10.2	607	7.5	2.0*	(1.4 ; 2.7)	0.4	(-0.2 ; 1.1)	C18-21
Hígado	305	6.4	311	4.7	317	5.7	372	4.5	-0.2	(-0.8 ; 0.5)	-1.5*	(-2.1 ; -0.8)	C22
Páncreas	180	3.8	235	3.5	252	4.5	300	3.7	-0.6	(-1.3 ; 0.2)	-1.2*	(-2 ; -0.5)	C25
Pulmón	714	14.8	531	8.0	799	14.4	602	7.3	-1.8*	(-2.2 ; -1.4)	-0.8*	(-1.3 ; -0.3)	C33-34
Melanoma de piel	105	2.1	102	1.4	152	2.7	126	1.6	2.2*	(1.2 ; 3.2)	1.2	(-0.1 ; 2.5)	C43-44
Mama	8	0.2	904	14.1	7	0.1	1,055	13.8			0.1	(-0.4 ; 0.6)	C50
Cérvix uterino			471	7.4			487	6.5			-3.9*	(-4.3 ; -3.5)	C53
Cuerpo uterino			115	1.7			138	1.9			-2.3*	(-3.5 ; -1.1)	C54-C55
Ovario			297	4.7			302	4.0			-0.6	(-1.5 ; 0.3)	C56
Próstata	847	16.7			1,012	17.4			-0.1	(-0.5 ; 0.4)			C61
Vejiga	100	2.0	58	0.8	113	2.0	75	0.9	-1.2*	(-2.4 ; -0.1)	-2.5*	(-3.9 ; -1.1)	C67
Linfoma y mieloma múltiple	256	5.3	237	3.8	330	6.0	290	3.7	-0.8*	(-1.5 ; 0.0)	-1.1*	(-1.9 ; -0.3)	C81-C90,C96
Leucemia	259	5.1	286	4.6	270	4.6	246	3.5	-0.6	(-1.2 ; 0.0)	-0.6	(-1.5 ; 0.3)	C91-95
Otros sitios	1,003	206	1,159	17.8	1,191	2.1	1,316	1.7	-0.7*	(-1.1 ; -0.3)	-1.2*	(-1.6 ; -0.9)	**
Todas las localizaciones	5,180	106	5,932	90.7	6,030	10.7	6,731	85.9	-0.9*	(-1.1 ; -0.6)	-1.3*	(-1.4 ; -1.1)	C00-C97

Número de casos (n);

ASR: Tasa de mortalidad estandarizada por edad (ASR, por su sigla en inglés).

APC: Por su sigla en inglés Annual Percent Change. APC es calculado para periodo 1984-2015.

*El APC es significativamente diferente de cero (p<0.05).

**C17, C23, C24, C26-C32, C37-C41, C45-C49, C51, C52,C57-C60, C62-C66, C68-C80, C97

El cáncer fue una de las principales causas de muerte en Cali, fue la tercera causa tras la mortalidad por lesiones y por enfermedades cardiovasculares que supone el 26.0% de las defunciones (48.8% en hombres y 51.2% en mujeres). La muerte por lesiones intencionales y no intencionales representa 20.1%.

En contraste con el número de defunciones, las tasas estandarizadas de mortalidad por cáncer para todas las localizaciones combinadas fueron mayores entre los hombres 107.0 que entre las mujeres 85.9. Los cánceres de estómago, pulmón, colon/recto, mama y próstata fueron las fueron las principales causas de muerte por cáncer, en conjunto representan aproximadamente la mitad de todas las muertes por cáncer (47.9%). 

Con base en las tasas de mortalidad estandarizadas por edad, el cáncer de próstata (ASR: 17.4), encabezó la mortalidad por tumores en varones en el quinquenio 2011-2015, seguidos del cáncer de estómago (ASR: 14.4), pulmón (ASR: 14.4), colon y recto (ASR: 10.2) y linfomas (ASR: 6.0). El cáncer de mama fue la principal causa de muerte en mujeres (ASR: 13.8), seguido del cáncer de estómago (ASR: 8.2), colon y recto (ASR: 7.5), pulmón (ASR: 7.3) y cuello uterino (ASR: 6.5). 

### 3. Cambios en la morbilidad y mortalidad por cáncer

Las Tablas 1 y 2 muestran el APC que representa el promedio porcentual de aumento o disminución anual de las tasas de incidencia y mortalidad por cáncer durante los periodos 1962-2012 y 1984-2015, respectivamente. Al describir el cambio, se detectaron tres patrones bien definidos: aumento o disminución cuando el APC fue significativamente diferente de cero (valores de dos colas *p* <0.05); de lo contrario se utilizó el término estable o plana. 

Las tasas de incidencia para todas las localizaciones del cáncer aumentaron en los hombres un promedio anual de 0.6% (IC 95%: 0.4; 0.8) y permanecieron estables en las mujeres. En contraste, la mortalidad para todas las localizaciones del cáncer ha ido disminuyendo significativamente un promedio anual de 0.9% en los hombres; (IC 95%: -1.1; -0.6); y 1.3% en las mujeres, (IC 95%: -1.4; -1.1). 

#### Tendencia de las tasas de incidencia de cáncer (1962-2012)

Las TIEE de cáncer disminuyeron tanto en hombres como en mujeres en las siguientes localizaciones: cavidad oral y faringe, esófago, estómago, laringe, vejiga urinaria y leucemias de tipo no especificado. La disminución sólo se observó en hombres en cáncer de páncreas y en la enfermedad de Hodgkin; y en las mujeres en cáncer de cuello uterino. 

En hombres y mujeres, se encontró aumento de las tasas de incidencia de cáncer en colon/recto, melanoma de piel, linfoma no-Hodgkin y leucemia linfoide; sólo en mujeres en cáncer de mama y tiroides; y sólo en hombre en cáncer de hígado, próstata y testículo. 

En las mujeres no hubo cambio en el riesgo de morbilidad por cáncer de hígado, páncreas, pulmón, cuerpo uterino, ovario y linfoma de Hodgkin y en los hombres permanecieron estables las TIEE de cáncer de tiroides y leucemia mieloide. 

#### Tendencia de las tasas de mortalidad por cáncer (1984-2015)

La mortalidad por cáncer muestra una tendencia favorable, sólo hubo aumento de las tasas de mortalidad por melanoma y cáncer colon/recto en los hombres. En el resto de neoplasias, se encontró evidencias de disminución de las tasas de mortalidad para diez de las 17 principales localizaciones. La disminución se observó tanto en hombres y mujeres en cáncer de esófago, estómago, pulmón, vejiga urinaria, linfomas y mieloma múltiple; sólo en hombres, en cáncer cavidad oral y faringe; y sólo en mujeres, en cáncer de hígado, páncreas y los tumores del tracto femenino: cérvix y cuerpo uterino.

No hubo cambios en la mortalidad por leucemias en toda la población de Cali. En los hombres permanecieron estables las tasas de mortalidad por cáncer de hígado, páncreas y próstata; y en las mujeres no hubo cambios en las tasas de mortalidad por cáncer mama, colorrectal, ovario y melanoma. 

### 4. Supervivencia neta a cinco años

Para el análisis se incluyeron en total 38,671 pacientes diagnosticados con cáncer en el periodo comprendido entre los años 1995-2009. La distribución de las neoplasias malignas más frecuentes correspondió a cáncer de mama (17.7%), próstata (17.3%), estómago (13.1%) y colorrectal (9.4%), mientras que un número menor de registros fueron reportados para cáncer de hígado (2.2%), melanoma (1.8%), mieloma múltiple (1.5%) y linfoma de Hodgkin (1.0%). La edad mediana al diagnóstico en términos generales para el periodo considerado fue de 64 años. Por último, con relación al periodo de diagnóstico es claro que ha habido un incremento en el número de pacientes diagnosticados para el último periodo de estudio 2005-2009. 

La tendencia de la supervivencia neta para determinados tipos de cáncer por sexo y período de diagnóstico 1995-2009 se muestran en la Figura 1.

**Figura 1:** Cali, Colombia. Supervivencia neta estandarizada por edad a 5 años para las localizaciones más frecuentes por periodo de interés y sexo, entre 1995 y 2009.

La Figura 2 muestra la supervivencia neta a los 5 años estandarizada por edad durante tres períodos: 1995-1999, 2000-2004 y 2005-2009. Cuando se compara con períodos anteriores, los pacientes con diagnóstico de cáncer en el período más reciente (2005-2009) se observaron mejoras notables en la supervivencia neta de 5 años para la mayoría de los sitios de cáncer. Las proporciones de supervivencia mayor de cáncer en las mujeres podrían explicarse en parte por los tipos comunes de cáncer en las mujeres (por ejemplo, tiroides, mama y cánceres de cuello uterino) que tienen un pronóstico relativamente bueno. Cuando se examina por año de diagnóstico y localización del cáncer, en términos generales se evidencia que en el último periodo el cual comprende los años 2005-2009 se presentó un incremento en la supervivencia para la mayoría de las localizaciones a excepción de cáncer de estómago y colorrectal.

**Figura 2:** Cali, Colombia. Supervivencia neta estandarizada por edad a 5 años después del diagnóstico por periodo de interés, ambos sexos entre 1995 y 2009.

En el caso de cáncer de hígado no se lleva a cabo la estandarización por edad, debido a que algunas de las estimaciones de la supervivencia neta a 5 años específicas por edad necesarias para llevar a cabo la estandarización no estaban disponibles (supervivencia muy baja, al primer año de seguimiento fallecieron alrededor del 90% de los pacientes con cáncer en el hígado). 

Por otro lado, las estimaciones más altas de la supervivencia neta para el periodo 2005-2009 se presentaron en cáncer de tiroides (89.3%), próstata (83.2%), mama (74.4%) y melanoma (65.8%). En el caso de neoplasias hematolinfoides la supervivencia fue mejor en pacientes con linfoma de Hodgkin (64.4%) y no-Hodgkin (43.3%). En leucemias y mieloma múltiple la supervivencia fue más baja, con estimaciones para el periodo 2005-2009 de 28.1% y 22.8% respectivamente.

#### Cáncer infantil

El sistema de monitoreo de resultados clínicos de cáncer infantil (VIGICANCER) registró 1,428 niños (<15 años) y adolescentes (15.0 a 18.9 años) entre el 2010 y el 2016; diagnosticados y tratados en Cali. De estos, el 96.0% (n: 1,379) contribuyeron al seguimiento clínico (673 tumores hematolinfoides y 706 tumores sólidos). La supervivencia global a 5 años en niños fue de 52.0% (IC 95%: 48.7, 55.3) y en adolescentes de 44.0% (IC 95%: 35.4, 52.2) (Tabla 3). Por aseguramiento en salud la supervivencia a 5 años fue de 61.0% (IC 95%; 56.4, 65.3) para el POS contributivo y regímenes de excepción, 41.2% (IC 95%; 36.2, 46.1) para el POS subsidiado, 24.1% (IC 95%: 13.6, 36.3). 

**Tabla 3:** Cali, Colombia, 2009-2013. Frecuencia de casos de cáncer infantil y supervivencia global observada a 5 años según grupos de la ICCC-3 ^21^

Grupo ICCC-3	Niños	%	Adolescentes	%	SO	IC 95%	
**I**	453	39.7	87	30.3	48.3	43.3	53.2
**II**	112	9.8	34	11.9	74.1	65.5	81.0
**III**	209	18.3	36	12.6	40.2	32.4	47.7
**IV**	32	2.8	1	0.3	39.2	21.5	56.6
**V**	60	5.3	0	0.0	74.8	61.1	84.2
**VI**	56	4.9	0	0.0	62.5	47.3	74.5
**VII**	19	1.7	0	0.0	47.8	9.6	79.3
**VIII**	64	5.6	43	15.0	29.4	18.9	40.5
**IX**	57	5.0	17	5.9	32.3	19.9	45.3
**X**	39	3.4	27	9.4	61.2	45.2	73.8
**XI**	27	2.4	37	12.9	76.2	59.8	86.6
**XII**	13	1.1	5	1.7	56.3	20.9	80.9
**Total**	1,141	100.0	287	100.0	50.8	47.7	53.8

ICCC-3: Clasificación Internacional Cáncer Infantil versión 3 (ICCC-3, por su sigla en inglés)

SO: Supervivencia observada global a 5 años.

IC 95%: Intervalo de confianza al 95%

El grupo I de la clasificación internacional de cáncer infantil versión 3 (ICCC-3)^21^ fue el más frecuente tanto en los niños (39.7%) como en los adolescentes (30.3%). Del grupo I de la ICCC-3, las leucemias linfoblásticas agudas (LLA) representaron el 79.1%. El grupo II (linfomas y neoplasias reticuloendoteliales) representó el 11.4% en niños y el 14.1% en adolescentes. El 38.4% de los tumores de este grupo fueron linfomas de Hodgkin, 38.3% los no Hodgkin (sin Burkitt) y 23.3% los Burkitt. El grupo III de tumores del sistema nervioso central globalmente representó el 17.2% de todos los tumores del niño y del adolescente, siendo más frecuente en menores de 1 año (26.5%). El grupo IV (neuroblastoma y otros tumores del sistema nervioso periférico) representaron el 2.3% de todos los tumores de la niñez. El retinoblastoma (grupo V, 4.2%). Los tumores renales (grupo VI, 94% tumores de Wilms) representaron el 3.9% de todos los tumores del niño. El grupo VII representó el 1.3%. 

Los tumores óseos (grupo VIII) fueron el 6.9% de neoplasias del niño (5.6%) y adolescente (14.9%). El osteosarcoma (59.8 %) y el sarcoma de Ewing (32.7%) los más frecuentes. El grupo IX de sarcomas de tejidos blandos (5.0%) en niños, (5.9%) en adolescentes. El grupo de tumores germinales (grupo X) globalmente representó el 5.2%; en niños 3.4%, en adolescentes 9.4%. El grupo XI de tumores epiteliales en niños fue el 2.4% mientras que en adolescentes fue de 12.9%. En este grupo el tumor de tiroides fue el más frecuente 51.5%. El 1.3% fueron clasificados como neoplasias no especificadas (grupo XII).

## Discusión

El RPCC de la Universidad del Valle presenta la situación del cáncer en Cali, durante el periodo 2008-2015. Esta información es necesaria para que las autoridades sanitarias hagan las estimaciones del riesgo de cáncer para otras regiones de Colombia que carecen de registros de cáncer. Estas estadísticas complementan informes previos ^5^^,^^22^ y proporcionan un monitoreo continuo durante los últimos 55 años, que permite analizar la información de 50 años de incidencia (1962-2012), 30 años de mortalidad (1984-2015) y 15 años de supervivencia (1995-2009). 

Cali ha experimentado profundos cambios epidemiológicos y demográficos en el último medio siglo. La población se ha cuadriplicado y ha envejecido, y la expectativa de vida al nacimiento aumentó de 56.7 a 68.4 años ^13^^,^^15^; en la actualidad hay 33 adultos mayores por cada 100 menores de 15 años ^13^. La oferta de servicios de atención oncológica de Cali corresponde a la sexta parte de la capacidad instalada del país ^16^ para atender cada año alrededor de 9.000 casos nuevos de cáncer, la mitad de residentes permanentes y el resto; de pacientes procedentes del sur-occidente, una región que representa el 20% de la población colombiana ^13^. El 85% de los servicios oncológicos de Cali son privados ^16^, la atención no es integral y existen diversas barreras para el acceso a una atención oncológica de calidad. Las medidas gubernamentales orientadas a estabilizar el sistema de salud han sido infructuosas y existen evidencias de discriminación y selección por riesgo del paciente oncológico por parte de las entidades responsables de administrar los riesgos relacionados con la enfermedad ^23^. Como consecuencia, el desenlace clínico aun es desfavorable, porque los pacientes llegan en estados avanzados de la enfermedad y la supervivencia para la mayoría de las neoplasias malignas es baja en comparación a la observada en Europa y Estados Unidos ^24^^,^^25^. 

Coincidiendo con los cambios demográficos hay variaciones significativas en las tendencias, patrones y diferencias en las tasas de incidencia y en la mortalidad por cáncer. El aumento o disminución del riesgo de morbilidad y mortalidad por este grupo de enfermedades está determinado por diferentes factores. Hasta el momento, algunos son reconocidos y la mayoría se encuentran pendientes de identificar. Estos cambios pueden ser resultado de variaciones en la exposición de la población a diferentes factores de riesgo, mejor acceso a los servicios sanitarios y perfeccionamiento en las técnicas diagnósticas y de tratamiento ^26^^,^^27^.

Aunque persisten varias amenazas, la información disponible muestra evidencia de avances en el control de algunos tipos de cáncer en Cali. La mortalidad global por cáncer disminuyó de manera significativa en hombres y mujeres con un porcentaje de cambio anual del 1% durante el periodo 1984-2015 (APC: -0.9, IC 95%: -1.1 ; -0.6) y (APC: -1.3, IC 95%: -1.4 ; -1.1). La magnitud del descenso fue mayor en los pacientes con cánceres relacionados con el consumo de tabaco, con agentes infecciosos y en las neoplasias hematolinfoides donde se han logrado importantes avances terapéuticos, (Fig. 3).

**Figura 3:** Cali, Colombia. Tendencia en las tasas de cáncer en los últimos 55 años.

### Cáncer relacionado con agentes infecciosos

Las tasas de incidencia y mortalidad por cáncer de estómago y cuello uterino disminuyeron de manera significativa en los últimos 55 años (Fig. 3). El descenso es monotónico, continuo y comenzó antes de conocerse el rol prominente en los procesos de carcinogénesis del *Helicobacter pylori* ^28^ *y* el Virus del Papiloma Humano (VPH) ^29^. Estos cambios no se relacionan con intervenciones específicas contra estos agentes infecciosos, son el resultado de progresos en el desarrollo y mejoramiento de las condiciones sanitarias. El desarrollo económico determinó cambios en los estilos de vida y modificaciones de los factores de riesgo conocidos de cáncer gástrico. La refrigeración facilita el consumo de alimentos frescos y limita el uso de métodos de conservación de alimentos basados en químicos (salazón, desecación, ahumado, acidificación). 

En pleno siglo XXI el cáncer gástrico aún representa una gran carga social en Cali y en Colombia porque ocasiona el mayor número de defunciones por cáncer ^3^^,^^5^^,^^22^. La enfermedad es mortal cuando se descubre clínicamente porque el diagnóstico se hace en estados avanzados. Durante el periodo 1995 a 2009, la supervivencia neta a 5 años de los pacientes con cáncer gástrico en Cali fue inferior al 20%, con una fracción de curación del 15% y un tiempo medio de supervivencia de 6 meses para los casos no curados ^22^; resultados similares se observan en Chile y Costa Rica. Pero en otros países de Latinoamérica con igual o menor desarrollo las estimaciones de supervivencia son comparables a las observadas en Estados Unidos y Europa que están alrededor del 30%. Es probable que las estimaciones de supervivencia al cáncer gástrico en Ecuador, Costa Rica y Cuba se encuentren sobrestimadas ^24^^,^^25^. 

A pesar del descenso continuo de la incidencia y mortalidad por los tumores relacionados con agentes infecciosos; las tasas aún se mantienen altas y el número de casos continúa en aumento por el envejecimiento y crecimiento de la población ^30^. Es muy probable que esta tendencia al descenso se mantenga y continúen su curso sin intervención adicional; pero, en condiciones naturales, podría durar muchas décadas, quizás siglos, hasta alcanzar los valores observados actualmente en Estados Unidos y Europa. Es prioritario implementar medidas adicionales para acelerar el descenso, mejorar la supervivencia y lograr su control ^30^.

Las perspectivas para el control del cáncer gástrico son inciertas porque son insuficientes los avances terapéuticos, los resultados pre-clínicos de los esfuerzos para el desarrollo de vacunas contra *H. pylori*, han sido decepcionantes ^31^; y la detección temprana del cáncer gástrico en Latinoamérica ha mostrado resultados discordantes ^32^^,^^33^ y baja costo-efectividad ^34^. Teniendo en cuenta que durante las primeras etapas del tumor es clínicamente silente, una alternativa para su control es la prevención primaria mediante la erradicación de la infección por *H. pylori* que puede reducir el riesgo de desarrollar cáncer gástrico en personas sin lesiones precursoras ^30^^,^^34^^,^^35^. Es necesario desarrollar monoterapias para facilitar la adherencia al tratamiento antibiótico y pruebas no invasivas que identifiquen las afecciones gástricas premalignas para estratificar el riesgo de los pacientes. La detección simultánea de pepsinógenos séricos y anticuerpos contra *H. pylori* ha conseguido este objetivo en el Japón ^35^^,^^36^. Esta estrategia no ha sido validada de manera adecuada en Latinoamérica y continúa siendo una opción que requiere exploración con metodología válida.

El panorama es diferente y favorable para las mujeres con cáncer de cuello uterino (CaCu), la tasa de mortalidad por CaCu en Cali, está cercana a la meta del PDCC ^4^, pero aún es tres veces más alta en comparación con Estados Unidos y Europa; donde el riesgo de CaCu es la mitad del observado en Cali. Las tasas de incidencia y mortalidad por CaCu han disminuido por múltiples razones, entre otras se incluyen el descenso en las tasas de fecundidad, la mejoría en las condiciones socio-económicas y el establecimiento de un programa en toda la ciudad para prevenir el cáncer de cuello uterino a través del uso generalizado de la citología vaginal ^29^^,^^37^.

El conocimiento que la infección por ciertos genotipos de (VPH) es una causa necesaria del CaCu determina dos nuevos frentes para su prevención en el actual PDCC. A partir del 2012, se establecen los lineamientos nacionales para la vacunación contra el VPH y se logra una cobertura del 80% que desafortunadamente es ahora inferior al 10% como consecuencia de un episodio mal manejado de reacción psicogénica masiva en Carmen de Bolívar, una región rural Colombiana. Dicha reacción fue supuestamente asociada a la vacuna contra el VPH ^38^. Para aumentar la precisión de la detección del CaCu, se inicia la tamización con pruebas que detecten los genotipos de alto riesgo del VPH. Se espera lograr una cobertura del 80% de la población objeto en el 2021 ^4^. Estas medidas son esenciales para acelerar el control de esta enfermedad porque la supervivencia neta a 5 años de las mujeres con CaCu en Cali es 57%, diez puntos porcentuales por debajo de la observada en los países afluentes ^24^^,^^25^. 

### Cáncer relacionados con actividades de tamización de oportunidad

El cáncer de próstata y mama son la primera causa de morbilidad por cáncer en hombres y mujeres de Cali ^5^. En Colombia no hay un programa organizado de cribado y su control se basa en actividades específicas de tamización de oportunidad implementadas en los ochenta y noventa del siglo pasado. La mamografía, tacto rectal y PSA permitieron detectar los casos de enfermedad que antes eran desconocidos y contribuyeron a aumentar las tasas de incidencia hasta el primer quinquenio del siglo 21 y desde entonces, comenzó su descenso. Gran parte, pero no todo el aumento, puede ser en los estadios tempranos de la enfermedad. Una vez estabilizado el uso de las pruebas de detección, las tasas tienden a estabilizarse siempre y cuando otros factores causantes de la enfermedad no hayan cambiado. Estos cambios fueron más evidentes en la población objeto de la tamización, el grupo de 50-69 años de edad, donde además hubo un punto de inflexión en la tendencia de la incidencia. Cambios similares se documentan en Costa Rica y Ecuador al final de la primera década del siglo XXI y fueron observados en Europa y Estados Unidos con 20 años de anterioridad. 

La mortalidad por cáncer de próstata disminuyó de manera consistente desde 1984 con porcentaje medio anual del 2%; el descenso se produce antes de lo esperado para poder atribuirlo exclusivamente a las actividades de tamización (Fig. 3). Un factor influyente y talvez determinante es la evolución de los tratamientos con intención curativa, es probable que el uso del PSA y el tacto rectal hayan contribuido a que se mantenga y consolide esta tendencia ^39^. En contraste, la mortalidad por cáncer de mama permaneció estable durante el periodo estudiado (Fig. 3). 

Estados Unidos y Europa han logrado grandes avances en el control del cáncer de próstata y mama. A pesar de las altas tasas de incidencia (ASR: 119.8 y ASR: 124.9, respectivamente); la supervivencia neta a 5 años se encuentra alrededor del 98.9% y 89.7%; y las tasas mortalidad alrededor de (ASR: 20.1, ASR: 21.2), respectivamente ^11^^,^^24^. En Cali la supervivencia neta a 5 años para las mismas neoplasias es 83.2% y 74.4%; y las tasas de mortalidad alrededor de (ASR: 17.4 y ASR:13.8), respectivamente. La existencia de una brecha de 15 puntos porcentuales en la supervivencia neta a 5 años en una población donde las tasas de incidencia son la mitad de las observadas en los países afluentes ^40^, sugiere que el diagnóstico de los casos se hace en etapas tardías y/o que los tumores son más agresivos. Estas inquietudes ameritan ser resueltas mediante investigaciones específicas. 

La incidencia y mortalidad por cáncer colo-rectal continua en ascenso en hombres y mujeres de Cali porque hasta el momento son incipientes las actividades de tamización y los factores de riesgo son difíciles de intervenir y/o no se encuentran claramente identificados ^41^; es prioritario impulsar un programa organizado de cribado para revertir la tendencia actual. Hasta tanto ocurra esta intervención la atención oncológica debe orientarse al diagnóstico temprano de los casos sospechosos. 

### Cáncer relacionado con el consumo de tabaco

La tendencia de la incidencia de cáncer de pulmón se correlaciona con los patrones históricos de prevalencia del consumo de cigarrillos y existe suficiente evidencia de relación causal entre su consumo y varios tipos de cáncer. Las variaciones en las medidas de ocurrencia del cáncer relacionado con el consumo de tabaco en Cali, se interpretan como un ejemplo exitoso de control del cáncer relacionado con una muy fuerte campaña antitabaco que tiene lugar en Colombia desde los años setenta y que se ha consolidado en el siglo 21. Las tasas de incidencia de cáncer de pulmón para ambos sexos en Cali reflejan el aborto de una epidemia relacionada con el tabaquismo que comenzó en los setenta y fue interrumpida alrededor de los ochenta ^5^^,^^42^^,^^43^. Desde entonces, hay disminución significativa de la incidencia y mortalidad por cáncer relacionado con el consumo de tabaco: cavidad oral y faringe, esófago, páncreas, pulmón y vejiga urinaria. La disminución es consistente en cavidad oral y pulmón donde se observa en hombres y mujeres. 

### Estimaciones de supervivencia neta

La vigilancia de la supervivencia del cáncer es importante para las organizaciones sanitarias, la sociedad civil y las agencias de investigación porque sirve para formular estrategias y para dar prioridad a las medidas de control del cáncer, y para evaluar la eficacia, y la relación costo-eficacia de esas estrategias ^1^. 

En los comienzos del siglo 21 iniciamos la vigilancia de las tendencias en la supervivencia del cáncer en Cali. Se estimó la supervivencia relativa sin estandarizar por edad, para 16,064 pacientes con diagnóstico de cáncer de próstata, mama, colorrectal, cuello uterino, estómago y pulmón durante 1995-2004 ^22^. Con el presente estudio se extiende la cobertura a 38,671 pacientes diagnosticados con un cáncer primario invasivo en catorce localizaciones que representan alrededor del 71.8% de la carga global de cáncer en Cali en el período de 15 años 1995-2009. Además, se mejora la precisión de las estimaciones previas, con la implementación de un nuevo método: la supervivencia neta ^44^^-^^46^ a 5 años estandarizadas por edad y ajustada por la mortalidad de base en Cali, por edad (un año), sexo y año calendario.

Coincidiendo con la implementación del nuevo sistema de salud en la década de los noventa, la supervivencia mejoró para la mayoría de las neoplasias en el primer quinquenio del siglo 21 en comparación con el periodo 1995-1999. Esta tendencia se estancó en el quinquenio 2005-2009 y la supervivencia neta a 5 años es similar con la encontrada en Argentina, Chile, Ecuador y Costa Rica y muy baja en comparación con la observada en los países afluentes ^24^. 

### Certificación de la mortalidad por cáncer

La información de mortalidad por cáncer en hígado, pulmón, cerebro y huesos debe interpretarse con precaución, porque en estos sitios, es frecuente la ocurrencia de metástasis. En estas localizaciones hubo casos 2,447 nuevos y 450 defunciones por cáncer. Se comparó la codificación de la topografía realizada por la oficina de estadísticas vitales y el registro de cáncer. Con los criterios de Landis ^47^, la concordancia para la codificación de los casos de cáncer de hígado, hueso y pulmón fue considerable (Kappa: 0.64, 0.67 y 0.79, respectivamente) y casi perfecta para los tumores malignos de SNC (Kappa: 0.90). De todas maneras, hay que tener en cuenta que el 45% de las defunciones codificadas como cáncer de hígado corresponden a metástasis. En el registro de cáncer había evidencias del sitio primario en otra localización. Esta proporción alcanzó las proporciones de 46%, 15% y 10% para las localizaciones de huesos, pulmón y SNC.

### Cáncer infantil

En el mundo se diagnostican alrededor de 200 000 casos nuevos de cáncer infantil ^48^, de los cuales 84% ocurren en países de medianos y bajos recursos ^49^^,^^50^. Considerando que el cáncer infantil no es prevenible, la supervivencia es la medida más relevante para evaluar los esfuerzos realizados para el control de este grupo de neoplasias en la población. La supervivencia global a 5 años del cáncer infantil en Cali (51%) esta 26% a 32% por debajo de los resultados reportados en países afluentes (77% a 83%) ^51^^,^^52^. Esto significa, que si en Colombia se tratan alrededor de 1,500 a 1,600 niños con cáncer al año, 765 a 816 mueren en los 5 años posteriores al diagnóstico. De estos, 390 a 512 muertes anuales serían evitables. Esta brecha de supervivencia se mantiene en todos los grupos de neoplasias, excepto en el linfoma de Hodgkin (88% vs 95%). Esta brecha es menor para el régimen contributivo (16%) que para el subsidiado (36%) o para la población pobre no asegurada (53%). Estas disparidades en los resultados clínicos se presentan a pesar de que el cubrimiento del aseguramiento de los niños con cáncer se supone igual entre los diferentes grupos, y que en Cali, los protocolos de quimioterapia utilizados son iguales entre estos grupos. La pérdida de eficacia de los tratamientos de quimioterapia tiene que ver principalmente por la intensidad de estos (dosis y tiempo). Esta intensidad del tratamiento se ve muy afectada por los retrasos en la colocación de los ciclos de quimioterapia, la baja adherencia y en su forma extrema el abandono del tratamiento. Las causas que conllevan a esta pérdida de intensidad son multifactoriales y tienen que ver tanto con el paciente, las familias, como con los prestadores y la integralidad del sistema de salud de forma global. La otra causa general de pérdida de eficacia de estos tratamientos son las muertes atribuibles al tratamiento y no la enfermedad. Esta es muy dependiente de los retrasos en el acceso de los niños al diagnóstico preciso, oportuno y a la calidad de atención de las unidades de oncología pediátrica de la ciudad. Las barreras de acceso también contribuyen a los malos resultados de tumores dependientes del estadio para lograr su curación; siendo el ejemplo más claro el retinoblastoma. En el contexto Latinoamericano, Argentina reporta 61.7% de supervivencia global a 3 años de los niños con cáncer ^53^. Esto representa una brecha global de supervivencia a 3 años con lo reportado en Cali del 5.6% (61.7 vs 56.1). 

### Limitaciones

RPCC no realiza seguimiento activo a los adultos, y Cali carece de estadísticas confiables sobre la migración de la población ^13^. El RPCC dispone de la información sobre la causa de muerte mediante los certificados de defunción, pero en algunos casos puede ser difícil determinar si el cáncer es la causa básica de la muerte. 

### Fortalezas

El RPCC ha participado en muchos otros estudios colaborativos, ha sido asesor del gobierno colombiano en la evaluación de PBCR en el país y sus datos han contribuido significativamente a diferentes aspectos del control del cáncer en Colombia. El trabajo colaborativo con la SSPM de Cali facilita el acceso a la información de la mortalidad general y por cáncer; y permite tener una fuente independiente de verificación de los casos nuevos de cáncer. El acceso al sistema de información del Ministerio de Salud (SISPRO) y a las bases de datos de aseguramiento proporciona una actualización permanente del estado vital y fecha de último contacto.
